# Oxidative balance score: a potential tool for reducing the risk of colorectal cancer and its subsites incidences

**DOI:** 10.3389/fendo.2024.1397512

**Published:** 2024-04-30

**Authors:** Yu Chang, Fan Li, Zhi Wang, Qi Zhao, Zhaodi Wang, Xiaoping Han, Zifeng Xu, Chanjiao Yu, Yue Liu, Shiyu Chang, Hongyan Li, Sileng Hu, Yuqin Li, Tongyu Tang

**Affiliations:** ^1^Department of Gastroenterology, The First Hospital of Jilin University, Changchun, China; ^2^Norman Bethune Health Science Center, Jilin University, Changchun, China

**Keywords:** oxidative balance score, colorectal cancer, UK biobank, mediation analysis, antioxidant

## Abstract

**Background:**

The Oxidative Balance Score (OBS) is commonly used to assess oxidative stress and provides a comprehensive evaluation of dietary and lifestyle-related exposures. However, there is limited research on the association between OBS and colorectal cancer (CRC), its subsites, and complications. The objective of this study was to assess the relationship between OBS and the risk of CRC, its subsites, and common complications in a large prospective cohort study.

**Methods:**

We included data from 175,808 participants in the UK Biobank data sample repository from 2006 to 2010. We evaluated OBS using a scoring system based on 22 dietary and lifestyle factors. Multiple adjustments, including multivariate Cox proportional hazard regression, gender stratification, subgroup analysis, and sensitivity analysis, were performed to fully explore the relationship between OBS and CRC, its subsites, and complications. The mediation analysis was conducted to investigate whether serum albumin, uric acid, and neutrophil levels mediate the relationship between OBS and CRC.

**Results:**

After adjusting for potential confounding factors, a significant negative correlation was found between OBS and the risk of CRC and its subsites (proximal colon cancer, distal colon cancer, and rectal cancer). This correlation was particularly pronounced in male CRC patients. Serum albumin, uric acid, and neutrophil count, which are biomarkers, were found to have a significant mediating effect between OBS and CRC.

**Conclusion:**

Our study suggests that higher exposure to antioxidants assessed through OBS (diet and lifestyle rich in antioxidants) may decrease the occurrence of CRC and its subsites.

## 1 Introduction

Colorectal cancer (CRC) is the third most common cancer worldwide, with an increasing incidence rate year by year (1). The prevalence of CRC varies in different regions and is influenced by both unmodifiable factors such as age, gender, and family history, and modifiable factors such as diet and lifestyle. Smoking, alcohol consumption, and high-fat diet are believed to be positively associated with the occurrence of CRC. Conversely, high intake of dietary fiber (fruits, vegetables, whole grains, etc.) and vitamins (vitamin C, D, E) has been found to have a positive protective effect against CRC (2, 3).

Oxidative stress is often considered as a balance between oxidation and antioxidant defense, and the excessive generation of reactive oxygen species (ROS) and the decrease in antioxidant defense capacity are often regarded as initiators and promoters of CRC. Oxidative stress can induce carcinogenesis through mechanisms such as inducing oxidative damage to deoxyribonucleic acid (DNA), protein oxidation, and inflammatory damage to intestinal epithelial cells (4, 5). Antioxidant enzymes and nutrients are believed to play a role in preventing carcinogenesis. However, the impact of individual oxidative balance exposure on carcinogenesis is difficult to measure, which may be closely associated with the combined action of various prooxidants and antioxidants (6). Therefore, in this study, the oxidative balance score (OBS) was used as an evaluation index for combined exposure to antioxidants and prooxidants, providing different assessment criteria for diet and lifestyle. Higher OBS scores represent greater exposure to antioxidants. Previous studies have shown a negative correlation between higher OBS and the occurrence of colorectal cancer (7, 8). However, few studies have explored the correlation between OBS and CRC, as well as its subsites, and the correlation between OBS related to diet and lifestyle and CRC in different genders.

The aim of this study was to investigate the association between OBS and CRC and its subsites (proximal colon cancer, distal colon cancer and rectal cancer) as well as complications (metastasis and abdominal pain) in a large UK Biobank follow-up cohort. Furthermore, we explored whether serum albumin, uric acid, and neutrophil levels mediated the associations mentioned above. Our findings will provide new evidence for the role of oxidative balance in CRC, as well as its subsites, and its related complications. These findings may have implications for early prevention and treatment of CRC in clinical practice.

## 2 Methods

### 2.1 Study population

The UK Biobank is a large prospective cohort study that recruited approximately 500,000 participants aged 40-69 from various regions of the UK between 2006 and 2010. Baseline data were collected at this time, followed by four large-scale follow-up visits and additional data collection. The database collected information from various modules, including sociodemographic, dietary, blood and urine specimens, environmental factors, and has been tracking and recording decades of health and medical records. Furthermore, since recruitment, the study has established links with cancer and mortality registries to conduct a tracking investigation on cancer incidence and mortality rates among participants. Approval for accessing patient records for recruitment was obtained from the National Health and Social Care Information Governance Committee for England and Wales, as well as the Community Health Index Advisory Group for Scotland. This biobank has obtained approval from the North West Multi-Centre Research Ethics Committee, and all participants have signed informed consent forms using touchscreen signature capture devices (9).

**Figure 1:**
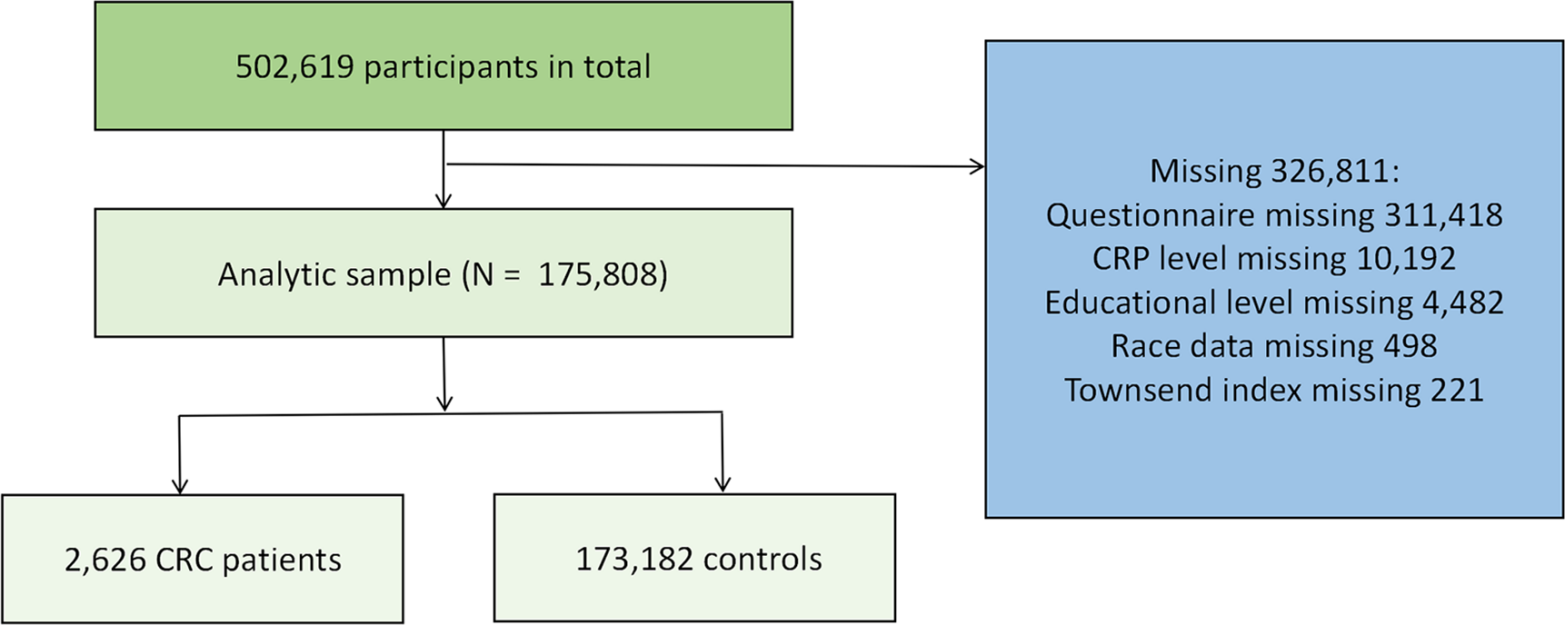
The sample sample selection and analysis flowchart.

The dataset included information from a total of 502,619 male and female participants. During the recruitment phase, 311,418 participants with incomplete questionnaire data and were unable to calculate the OBS were excluded. Furthermore, patients with missing data (including race, education level, CRP levels, and Thompson Index (TSI) were excluded. Ultimately, a total of 175,808 participants were included in this study. Further specific details are available in **Figure 1**.

### 2.2 Assessment of CRC

Incidence of cancer and mortality cases in the UK Biobank were primarily obtained by linking to routine data from the National Health Service in the UK. Cancer types were coded using the International Classification of Diseases (ICD-10), while histological and morphological information of tumors were coded using ICD-O-3. Participants were followed up from the time of recruitment, and the analysis was initiated from the date when eligible participants were first registered as having cancer. The study exit date, death date, or last follow-up date was December 31, 2014. The endpoint of the analysis was the first diagnosis of CRC (ICD-10 codes C18-C20) or the primary underlying cause of death due to CRC. Proximal colon cancer was defined as tumors occurring in the cecum, appendix, ascending colon, hepatic flexure, transverse colon, or splenic flexure (C18.0-C18.5). Distal colon cancer was defined as tumors occurring in the descending colon (C18.6) or sigmoid colon (C18.7). Rectal cancer included tumors occurring at the rectosigmoid junction (C-19) and rectum (C-20).

### 2.3 Measurement of OBS

Four categories of data were obtained and collected using a food frequency questionnaire (FFQ), including dietary pro-oxidants (total fat, iron, polyunsaturated fatty acids, saturated fatty acids, meat intake) and lifestyle pro-oxidants (smoking, alcohol consumption, BMI). Dietary antioxidants (calcium, magnesium, vitamin B6, vitamin B12, vitamin C, vitamin D, vitamin E, total folate, carotenoids, dietary fiber, retinol, vegetable intake) and lifestyle antioxidants (tea consumption and physical activity) were also collected. The cumulative OBS score was derived by summing the assigned values (0, 1, 2) based on the predefined tertiles, and participants were further divided into groups based on gender. Participants in the lower tertiles were assigned a value of 0, while those in the higher tertiles were assigned a value of 2. Consequently, a score of 0 represented a low antioxidant level, while a score of 2 indicated a high antioxidant level. Therefore, a higher OBS score indicated a more significant exposure to antioxidants. In smoking, alcohol consumption, and meat intake, non-consumption was defined as 2, and values of 0 or 1 were assigned based on binomial distribution grouping. Meat intake was calculated by summing the intake of beef, lamb, and pork, while vegetable intake was calculated by summing the intake of raw and cooked vegetables. Physical activity was obtained by summing the weekly MET minutes of light, moderate, and vigorous physical activities. For more specific details, please refer to **Supplementary Table 1**.

### 2.4 Covariates

In this study, a Cox proportional hazards model was constructed to adjust for potential confounding factors that may influence the association between OBS and colorectal cancer. The adjusted covariates included age, race (European, Asian, African, Chinese, Mixed-race, Others), Townsend deprivation index, education achievement index, BMI, high-sensitivity CRP, dietary energy intake, and NSAIDs medication usage. The education achievement index data were obtained from two domains: “child and adolescent” and “adult skills,” reflecting both the mobility and stock of educational disadvantages within a region. Dietary energy intake data were calculated based on recommended data from dietary questionnaire surveys, and NSAIDs medication usage data were obtained from self-reported survey questionnaires.

### 2.5 Statistical analyses

Continuous variables in this study were presented as mean (± standard deviation). Categorical variables were presented as sample size (percentage). Continuous OBS scores were categorized based on quartiles (Q1: < 25th percentile, Q2: 25th-50th percentile, Q3: 50th-75th percentile, Q4: 75th-100th percentile). In our study, grouping OBS scores into quartiles was aimed at better understanding the distribution of OBS scores among participants and further investigating the relationship between OBS scores and other variables. Wilcoxon rank sum test was used to describe the differences in baseline variable characteristics between quartile groups of continuous OBS scores. Rao-Scott χ2 test was used to describe the percentage differences of categorical variables. Multiple Cox proportional hazards models were established to explore the correlation between OBS and the occurrence of CRC and its subsites (proximal colon cancer, distal colon cancer and rectal cancer). Model 1: Adjusted for age, race, educational attainment, and Townsend deprivation index; Model 2: In addition to Model 1, adjusted for dietary energy intake; Model 3: In addition to Model 2, adjusted for plasma CRP concentration and NSAIDs medication usage. Furthermore, we conducted subgroup analysis stratified by gender to further investigate the correlation between the two in the three models mentioned above. In this study, we explored the correlation between continuous OBS and the occurrence of complications in CRC (colorectal cancer metastasis, proximal colon cancer metastasis, abdominal pain in proximal colon cancer, abdominal pain in distal colon cancer) using the aforementioned three models. We employed logistic regression models adjusted for the variables in Model 3 to investigate the correlation between continuous variables including serum albumin, uric acid, and neutrophil levels and the occurrence of CRC. In addition, sensitivity analysis was conducted by performing leave-one-out analysis to assess the contribution of each score, and OBS was further divided into diet OBS and lifestyle OBS. The relationship between OBS and CRC occurrence was separately explored in gender subgroups. Mediation analysis was performed to determine whether serum albumin, uric acid, and neutrophil levels mediated the relationship between OBS and CRC. Indirect effect (IE) refers to the influence of serum albumin, uric acid, and neutrophil levels on CRC through OBS. Direct effect (DE) represents the influence of OBS on CRC after controlling for serum albumin, uric acid, and neutrophil levels. Significantly positive IE indicates the presence of a significant mediating effect. All statistical analyses and graphical presentations were performed using R Project for Statistical Computing (version 4.2.3). All statistical tests were two-tailed, and a p-value < 0.05 was considered statistically significant.

## 3 Results

### 3.1 Baseline characteristics

We grouped the participants based on quartiles of OBS and summarized the baseline characteristics of the total population and each group of OBS. The variability in the number of subjects in each group primarily stems from the uneven distribution of OBS scores in the actual data, with different densities across different score ranges. Hence, the unequal distribution of subjects across the four groups. Regarding the representation of quartile ranges in the table, we employed the convention of using closed on the left and open on the right intervals, such as [4,19), indicating that the first quartile range encompasses OBS scores equal to or greater than 4 and less than 19. A total of 175,808 participants were included in this study. The average age of the participants was 55.9 ± 8.0 years. Compared to participants in the lowest quartile of OBS, those in the highest quartile of OBS were more likely to be female, older, of European descent, with lower education levels, plasma CRP levels, NSAIDs medication usage and BMI, higher Townsend deprivation index and dietary energy intake, lower incidence and mortality rate of CRC. For more detailed baseline characteristics, see **Table 1**. We also compared the general characteristics between males and females. Compared to female participants, male participants were more likely to have lower OBS scores. They were also more likely to be older individuals with colorectal cancer and its related complications (obstruction and abdominal pain). Moreover, male participants had lower TDI scores, Levels of CRP and NSAIDs medication usage, higher level of education, BMI levels, and dietary energy intake. For more details, refer to **Supplementary Table 2**.

**Table 1:** Baseline characteristics of participants according to quartiles of oxidative balance score: comparison between the first and fourth quartiles.

	Overall N = 175,808	Quantile OBS groups				p-value
		Q1 [4,19) N = 40,379	Q2 [19,22) N = 35,004	Q3 [22,26) N = 49,209	Q4 [26,41) N = 51,216	
OBS, Mean (SD)	22.5 (5.0)	15.8 (2.1)	20.0 (0.8)	23.5 (1.1)	28.5 (2.2)	<0.001
Colorectal cancer, n (%)	2,626 (1.5%)	671 (1.7%)	502 (1.4%)	715 (1.5%)	738 (1.4%)	0.018
Proximal colon cancer, n (%)	786 (0.4%)	186 (0.5%)	164 (0.5%)	207 (0.4%)	229 (0.4%)	0.700
Distal colon cancer, n (%)	786 (0.4%)	216 (0.5%)	132 (0.4%)	223 (0.5%)	215 (0.4%)	0.008
Rectal cancer, n (%)	757 (0.4%)	196 (0.5%)	154 (0.4%)	202 (0.4%)	205 (0.4%)	0.200
CRC with obstruction, n (%)	275 (0.2%)	63 (0.2%)	60 (0.2%)	64 (0.1%)	88 (0.2%)	0.200
CRC with abdominal pain, n (%)	294 (0.2%)	81 (0.2%)	61 (0.2%)	60 (0.1%)	92 (0.2%)	0.068
Secondary Metastasis of CRC, n (%)	662 (0.4%)	184 (0.5%)	124 (0.4%)	176 (0.4%)	178 (0.3%)	0.084
Death of CRC, n (%)	509 (0.3%)	142 (0.4%)	97 (0.3%)	120 (0.2%)	150 (0.3%)	0.027
Sex, n (%)						<0.001
Female	94,044 (53%)	20,971 (52%)	18,392 (53%)	26,215 (53%)	28,466 (56%)	
Male	81,764 (47%)	19,408 (48%)	16,612 (47%)	22,994 (47%)	22,750 (44%)	
TDI, Mean (SD)	-1.6 (2.9)	-1.3 (3.0)	-1.6 (2.9)	-1.7 (2.8)	-1.8 (2.8)	<0.001
Missing	221	68	39	63	51	
Education, Mean (SD), score	11.7 (13.8)	13.3 (15.1)	11.7 (13.8)	11.2 (13.2)	11.0 (13.0)	<0.001
Missing	4,482	1,108	871	1,199	1,304	
Ethnicity, n (%)						<0.001
European	167,952 (96%)	38,319 (95%)	33,409 (96%)	47,162 (96%)	49,062 (96%)	
Mixed-race	1,054 (0.6%)	299 (0.7%)	197 (0.6%)	282 (0.6%)	276 (0.5%)	
Asian	2,488 (1.4%)	583 (1.4%)	526 (1.5%)	699 (1.4%)	680 (1.3%)	
African	2,082 (1.2%)	706 (1.8%)	410 (1.2%)	467 (1.0%)	499 (1.0%)	
Chinese	495 (0.3%)	90 (0.2%)	106 (0.3%)	142 (0.3%)	157 (0.3%)	
Others	1,239 (0.7%)	268 (0.7%)	253 (0.7%)	333 (0.7%)	385 (0.8%)	
Missing	498	114	103	124	157	
BMI, Mean (SD), kg/m2	26.9 (4.6)	28.3 (4.9)	27.1 (4.6)	26.6 (4.4)	25.8 (4.2)	<0.001
Age, Mean (SD), years	55.9 (8.0)	55.0 (8.0)	55.7 (8.0)	56.0 (7.9)	56.6 (7.9)	<0.001
CRP level, Mean (SD), mg/L	2.3 (4.0)	2.7 (4.3)	2.3 (3.9)	2.2 (4.0)	1.9 (3.7)	<0.001
Missing	10,192	2,377	2,009	2,868	2,938	
Daily energy intake, Mean (SD)	8,862.8 (3,036.6)	7,731.7 (2,481.1)	8,300.7 (2,718.7)	9,135.5 (2,997.0)	9,876.7 (3,285.3)	<0.001
NSAIDs medication usage, n (%)	59,478 (34%)	14,645 (36%)	12,147 (35%)	16,423 (33%)	16,263 (32%)	<0.001
Albumin, Mean (SD), g/L	45.4 (2.6)	45.3 (2.6)	45.4 (2.6)	45.4 (2.6)	45.4 (2.6)	<0.001
Missing	24,201	5,511	4,794	6,689	7,207	
Uric acid, Mean (SD), µmol	306.4 (79.1)	318.6 (82.0)	310.2 (79.7)	304.9 (78.3)	295.5 (75.7)	<0.001
Missing	10,035	2,334	1,988	2,808	2,905	
Neutrophils, Mean (SD), 10^9 cells/Litre	4.1 (1.4)	4.3 (1.4)	4.2 (1.4)	4.1 (1.3)	4.0 (1.3)	<0.001
Missing	6,810	1,608	1,447	1,849	1,906	

OBS, Oxidative Balance Score; TDI, Thomson Deprivation Index; CRC, Colorectal Cancer; SD, standard deviation; BMI, body mass index; CRP, C-reactive protein; Education refered to the age of Highest Level of Education.

### 3.2 Association between OBS and CRC and its subsites

As shown in **Table 2**, in the fully adjusted Model 3, there is a significant negative correlation between continuous OBS and the occurrence of CRC (HR 0.974, 95%CI 0.966-0.982, p<0.001), proximal colon cancer (HR 0.981, 95%CI 0.966-0.996, p=0.012), distal colon cancer (HR 0.966, 95%CI 0.951-0.981, p<0.001) and rectal cancer (HR 0.968, 95%CI 0.953-0.983, p<0.001). Compared to the Q1 of OBS, the risk of CRC decreased by 19.4% in Q2 (HR 0.806, 95%CI 0.714-0.909, p<0.001), 23.0% in Q3 (HR 0.770, 95%CI 0.689-0.861, p<0.001), and 28.7% in Q4 (HR 0.713, 95%CI 0.636-0.799, p<0.001). Compared to the Q1 of OBS, the risk of proximal colon cancer decreased by 19.5% in Q3 (HR 0.805, 95%CI 0.652-0.994, p=0.044) and 21.6% (HR 0.784, 95%CI 0.634-0.968, p=0.024). Compared to the Q1 of OBS, the risk of distal colon cancer decreased by 34.8% in Q2 (HR 0.652, 95%CI 0.520-0.816, p<0.001), 26.8% in Q3 (HR 0.732, 95%CI 0.600-0.893, p=0.002), and 36.5% in Q4 (HR 0.645, 95%CI 0.525-0.792, p<0.001). Compared to the Q1 of OBS, the risk of rectal cancer decreased by 26.2% in Q3 (HR 0.738, 95%CI 0.599-0.910, p=0.004) and 31.9% in Q4 (HR 0.681, 95%CI 0.550-0.841, p<0.001). More details can be obtained in **Table 2**.

**Table 2:** Association of oxidative balance score with colorectal cancer and subsites.

	Continue	Quantile				P for trend
		Q1	Q2	Q3	Q4	
CRC, HR (95% CI), P-value						
Model 1	0.979(0.971~0.987), <0.001	Ref.	0.807(0.718~0.908), <0.001	0.799(0.718~0.889), <0.001	0.757(0.681~0.842), <0.001	<0.001
Model 2	0.974(0.966~0.982), <0.001	Ref.	0.806(0.715~0.910), <0.001	0.771(0.689~0.863), <0.001	0.715(0.638~0.801), <0.001	<0.001
Model 3	0.974(0.966~0.982), <0.001	Ref.	0.806(0.714~0.909), <0.001	0.770(0.689~0.861), <0.001	0.713(0.636~0.799), <0.001	<0.001
Proximal colon cancer, HR (95% CI), P-value						
Model 1	0.985(0.972~0.999), 0.042	Ref.	0.951(0.769~1.176), 0.641	0.831(0.680~1.016), 0.071	0.823(0.676~1.002), 0.052	0.027
Model 2	0.981(0.967~0.996), 0.015	Ref.	0.953(0.765~1.188), 0.670	0.808(0.654~0.998), 0.048	0.789(0.639~0.975), 0.028	0.012
Model 3	0.981(0.966~0.996), 0.012	Ref.	0.951(0.763~1.185), 0.654	0.805(0.652~0.994), 0.044	0.784(0.634~0.968), 0.024	0.010
Distal colon cancer, HR (95% CI), P-value						
Model 1	0.973(0.959~0.987), <0.001	Ref.	0.663(0.532~0.826), <0.001	0.779(0.644~0.943), 0.010	0.694(0.573~0.841), <0.001	0.002
Model 2	0.966(0.951~0.981), <0.001	Ref.	0.652(0.521~0.816), <0.001	0.732(0.600~0.894), 0.002	0.646(0.526~0.793), <0.001	<0.001
Model 3	0.966(0.951~0.981), <0.001	Ref.	0.652(0.520~0.816), <0.001	0.732(0.600~0.893), 0.002	0.645(0.525~0.792), <0.001	<0.001
Rectal cancer, HR (95% CI), P-value						
Model 1	0.973(0.959~0.987), <0.001	Ref.	0.845(0.682~1.046), 0.122	0.773(0.633~0.943), 0.011	0.726(0.595~0.885), 0.002	0.001
Model 2	0.968(0.953~0.983), <0.001	Ref.	0.843(0.676~1.050), 0.127	0.739(0.600~0.911), 0.005	0.682(0.552~0.843), <0.001	<0.001
Model 3	0.968(0.953~0.983), <0.001	Ref.	0.842(0.676~1.049), 0.126	0.738(0.599~0.910), 0.004	0.681(0.550~0.841), <0.001	<0.001

The hazard ratio value of Q2-Q4 is based on the Q1 group as the reference. Model 1 was adjusted for age, race, educational attainment, and Townsend deprivation index. Model 2, building upon Model 1, adjusted for dietary energy intake; Model 3: In addition to Model 2, adjusted for plasma CRP concentration and NSAIDs medication usage. CRC, Colorectal Cancer; HR, Hazard Ratio.

### 3.3 Association between OBS and CRC and its subsites stratified by sex and race

As shown in **Table 3**, in male participants, in the fully adjusted Model 3, there is a significant negative correlation between continuous OBS and the occurrence of CRC (HR 0.967, 95%CI 0.956-0.977, p<0.001), proximal colon cancer (HR 0.974, 95%CI 0.954-0.995, p=0.016), distal colon cancer (HR 0.947, 95%CI 0.928-0.966, p<0.001) and rectal cancer (HR 0.978, 95%CI 0.959-0.997, p=0.026). Compared to Q1 of OBS, the risk of CRC decreased by 20.7% (HR 0.793, 95%CI 0.682-0.922, p=0.003) in Q2, 28.2% (HR 0.718, 95%CI 0.622-0.829, p<0.001) in Q3 and 34.1% (HR 0.659, 95%CI 0.568-0.766, p<0.001) in Q4. Compared to Q1 of OBS, the risk of distal colon cancer decreased by 28.2% (HR 0.560, 95%CI 0.422-0.741, p<0.001) in Q2, 38.0% (HR 0.620, 95%CI 0.483-0.796, p<0.001) in Q3 and 49.4% (HR 0.506, 95%CI 0.387-0.663, p<0.001) in Q4. Compared to Q1 of OBS, the risk of rectal cancer decreased by 22.8% in Q3 (HR 0.772, 95%CI 0.597-0.999, p=0.049). In female participants, in the fully adjusted Model 3, there is a significant negative correlation between continuous OBS and the occurrence of rectal cancer (HR 0.970, 95%CI 0.945-0.995, p=0.019). In model 2, compared to the Q1 of OBS, the risk of rectal cancer decreased by 30.9% in Q4 (HR 0.691, 95%CI 0.479-0.996, p=0.048). In model 3, compared to Q1 of OBS, the risk of rectal cancer decreased by 30.8% in Q4 (HR 0.692, 95%CI 0.480-0.997, p=0.048). More specific details can be seen in **Table 3**.

**Table 3:** Association between oxidative balance score and colorectal cancer and its subsites stratified by sex.

	Continue	Quantile				P for trend
		Q1	Q2	Q3	Q4	
CRC in male, HR (95% CI), P-value						
Model 1	0.968(0.958~0.978), <0.001	Ref.	0.789(0.682~0.913), 0.001	0.726(0.634~0.832), <0.001	0.665(0.579~0.764), <0.001	<0.001
Model 2	0.967(0.956~0.978), <0.001	Ref.	0.794(0.683~0.924), 0.003	0.720(0.624~0.831), <0.001	0.662(0.570~0.769), <0.001	<0.001
Model 3	0.967(0.956~0.977), <0.001	Ref.	0.793(0.682~0.922), 0.003	0.718(0.622~0.829), <0.001	0.659(0.568~0.766), <0.001	<0.001
Proximal colon cancer in male, HR (95% CI), P-value						
Model 1	0.980(0.961~0.999), 0.040	Ref.	1.122(0.851~1.481), 0.414	0.885(0.676~1.159), 0.376	0.802(0.609~1.055), 0.114	0.038
Model 2	0.975(0.955~0.996), 0.020	Ref.	1.119(0.842~1.488), 0.439	0.847(0.638~1.124), 0.249	0.761(0.567~1.022), 0.070	0.020
Model 3	0.974(0.954~0.995), 0.016	Ref.	1.114(0.837~1.481), 0.459	0.841(0.634~1.116), 0.231	0.752(0.560~1.011), 0.059	0.016
Distal colon cancer in male, HR (95% CI), P-value						
Model 1	0.949(0.931~0.967), <0.001	Ref.	0.559(0.425~0.736), <0.001	0.631(0.497~0.800), <0.001	0.512(0.398~0.658), <0.001	<0.001
Model 2	0.947(0.928~0.966), <0.001	Ref.	0.560(0.423~0.741), <0.001	0.621(0.484~0.796), <0.001	0.507(0.387~0.664), <0.001	<0.001
Model 3	0.947(0.928~0.966), <0.001	Ref.	0.560(0.422~0.741), <0.001	0.620(0.483~0.796), <0.001	0.506(0.387~0.663), <0.001	<0.001
Rectal cancer in male, HR (95% CI), P-value						
Model 1	0.976(0.958~0.994), 0.008	Ref.	0.786(0.603~1.024), 0.075	0.759(0.595~0.969), 0.027	0.759(0.595~0.967), 0.026	0.028
Model 2	0.978(0.959~0.998), 0.029	Ref.	0.793(0.602~1.044), 0.099	0.774(0.599~1.002), 0.051	0.790(0.607~1.028), 0.079	0.085
Model 3	0.978(0.959~0.997), 0.026	Ref.	0.791(0.601~1.042), 0.096	0.772(0.597~0.999), 0.049	0.786(0.604~1.023), 0.074	0.079
CRC in female, HR (95% CI), P-value						
Model 1	1.000(0.988~1.012), 0.994	Ref.	0.863(0.709~1.051), 0.143	0.965(0.810~1.150), 0.688	0.981(0.828~1.163), 0.824	0.798
Model 2	0.995(0.983~1.008), 0.481	Ref.	0.877(0.716~1.073), 0.202	0.951(0.792~1.143), 0.594	0.940(0.784~1.128), 0.507	0.755
Model 3	0.995(0.983~1.008), 0.477	Ref.	0.876(0.716~1.073), 0.202	0.951(0.792~1.142), 0.593	0.940(0.783~1.128), 0.505	0.752
Proximal colon cancer in female, HR (95% CI), P-value						
Model 1	0.995(0.974~1.015), 0.605	Ref.	0.761(0.545~1.062), 0.108	0.780(0.577~1.052), 0.104	0.861(0.648~1.144), 0.301	0.436
Model 2	0.994(0.972~1.017), 0.602	Ref.	0.772(0.543~1.096), 0.148	0.795(0.578~1.092), 0.157	0.869(0.639~1.183), 0.373	0.521
Model 3	0.994(0.972~1.016), 0.592	Ref.	0.771(0.543~1.096), 0.147	0.794(0.578~1.092), 0.156	0.868(0.637~1.181), 0.366	0.513
Distal colon cancer in female, HR (95% CI), P-value						
Model 1	1.014(0.992~1.037), 0.215	Ref.	0.961(0.658~1.403), 0.837	1.212(0.871~1.687), 0.255	1.230(0.891~1.699), 0.209	0.105
Model 2	1.007(0.983~1.031), 0.585	Ref.	0.941(0.640~1.382), 0.755	1.126(0.799~1.587), 0.498	1.151(0.819~1.619), 0.418	0.276
Model 3	1.006(0.983~1.031), 0.597	Ref.	0.940(0.640~1.381), 0.752	1.125(0.798~1.585), 0.502	1.149(0.817~1.615), 0.426	0.282
Rectal cancer in female, HR (95% CI), P-value						
Model 1	0.976(0.952~1.000), 0.048	Ref.	0.996(0.692~1.435), 0.985	0.840(0.594~1.189), 0.325	0.753(0.533~1.064), 0.107	0.064
Model 2	0.970(0.945~0.995), 0.019	Ref.	1.010(0.698~1.460), 0.959	0.796(0.556~1.140), 0.212	0.691(0.479~0.996), 0.048	0.022
Model 3	0.970(0.945~0.995), 0.019	Ref.	1.010(0.698~1.460), 0.958	0.796(0.556~1.140), 0.213	0.692(0.480~0.997), 0.048	0.022

The hazard ratio value of Q2-Q4 is based on the Q1 group as the reference. Model 1 was adjusted for age, race, educational attainment, and Townsend deprivation index. Model 2, building upon Model 1, adjusted for dietary energy intake; Model 3: In addition to Model 2, adjusted for plasma CRP concentration and NSAIDs medication usage. CRC, Colorectal Cancer; HR, Hazard Ratio. CI, confidence interval.

As shown in **Supplementary Table 3**, we stratified by age to further assess the correlation between OBS and CRC in different female cohorts above and below the age of 50. In multiple models adjusted for the same covariates as mentioned above, no significant correlation between OBS and CRC was found across different age groups in women. As shown in **Supplementary Table 4**, we further stratified by race to assess the association between OBS and CRC across different racial groups (European, Mixed, Asian, African, Chinese, and other races). In multiple models adjusted for the aforementioned variables, a significant inverse association between OBS and CRC was found only in the European ancestry population.

### 3.4 Correlation of OBS with CRC complications

As shown in **Table 4**, OBS is positively correlated with the risk of CRC metastasis in models 2 and 3. However, OBS is negatively correlated with the risk of proximal CRC metastasis. Furthermore, OBS is negatively correlated with the risk of abdominal pain caused by proximal CRC in models 1, 2, and 3. However, OBS is positively correlated with the risk of abdominal pain caused by distal CRC.

**Table 4:** Correlation of oxidative balance score with colorectal cancer complications.

Complications	Model 1		Model 2		Model 3	
	HR (95% CI)	P-value	HR (95% CI)	P-value	HR (95% CI)	P-value
Secondary Metastasis in CRC	1.043(1.000~1.089)	0.050	1.050(1.002~1.100)	0.041	1.052(1.004~1.102)	0.035
Secondary Metastasis in Proximal CRC	0.975(0.948~1.003)	0.079	0.967(0.938~0.996)	0.025	0.967(0.938~0.996)	0.025
Abdominal pain in Proximal CRC	0.962(0.932~0.994)	0.019	0.955(0.922~0.988)	0.008	0.954(0.922~0.988)	0.008
Abdominal pain in distal CRC	1.043(1.000~1.089)	0.050	1.050(1.002~1.100)	0.041	1.052(1.004~1.102)	0.035

Model 1 was adjusted for age, race, educational attainment, and Townsend deprivation index. Model 2, building upon Model 1, adjusted for dietary energy intake; Model 3: In addition to Model 2, adjusted for plasma CRP concentration and NSAIDs medication usage. CRC, Colorectal Cancer; HR, Hazard Ratio; CI, Confidence Interval.

### 3.5 Association between serum albumin, uric acid, neutrophils and CRC

As illustrated in **Table 5**, we classified the continuous levels of serum albumin, uric acid, and neutrophils into quartiles to explore the correlation between these three biomarkers and CRC. Compared to the Q1 group with respect to serum albumin concentration, the risks of CRC occurrence in the Q2, Q3, and Q4 groups decreased by 12.8% (OR 0.872 [0.784-0.971], p=0.012), 11.7% (OR 0.883, 95% CI [0.792-0.983], p=0.024), and 12.3% (OR 0.877, 95% CI [0.786-0.979], p=0.020). The continuous concentration of serum uric acid showed a significant positive correlation with CRC occurrence, with the risks of CRC in the Q3 and Q4 groups increasing by 22.0% (OR 1.220, 95% CI [1.095-1.360], p<0.001) and 37.9% (OR 1.379, 95% CI [1.239-1.535], p<0.001). The continuous levels of serum neutrophils showed a significant positive correlation with CRC occurrence, and the risk of CRC in the Q4 group was increased by 16.5% (OR 1.165, 95% CI [1.045-1.300], p=0.006). More details can be seen in **Table 5**.

**Table 5:** Association between serum albumin, uric acid, neutrophils and colorectal cancer.

Mediators	OR 95%CI, P-value				
	Continue	Q1	Q2	Q3	Q4
**Albumin**	1.000(0.999~1.001), 0.206	Ref	0.872(0.784~0.971), 0.012	0.883(0.792~0.983), 0.024	0.877(0.786~0.979), 0.02
**Uric acid**	1.001(0.999~1.001), <0.001	Ref	1.003(0.896~1.122), 0.957	1.220(1.095~1.360), <0.001	1.379(1.239~1.535), <0.001
**Neutrophils**	1.001(0.999~1.001), 0.033	Ref	1.068(0.960~1.187), 0.227	1.045(0.938~1.163), 0.423	1.165(1.045~1.300), 0.006

OR, Odds Ratio; CI, Confidence Interval.

### 3.6 Sensitivity analysis

**Table 6** presents the correlation between lifestyle OBS and dietary OBS with CRC occurrence in the overall participants and different gender subgroups. In the overall participants, both dietary OBS and lifestyle OBS were significantly and consistently negatively correlated with CRC occurrence. In the gender-stratified subgroup analysis, both dietary OBS and lifestyle OBS were significantly negatively correlated with male CRC occurrence. Additionally, we conducted a leave-one-out analysis, in which the contribution of each OBS component score was removed for reanalysis. The results indicated a significant negative correlation between OBS score and CRC occurrence in both the overall participants and male participants. However, no significant correlation was found between OBS score and CRC occurrence in female participants. More details can be seen in **Table 7**.

**Table 6:** Stratified study on the contribution of lifestyle and dietary intake to oxidative balance score.

	Lifestyle OBS	Nutrients intake OBS
CRC, HR (95% CI), P-value		
Model 1	0.935(0.919~0.952)<0.001	0.990(0.981~0.999)0.021
Model 2	0.941(0.925~0.958)<0.001	0.982(0.972~0.991)<0.001
Model 3	0.940(0.924~0.957)<0.001	0.982(0.972~0.991)<0.001
CRC in female, HR (95% CI), P-value		
Model 1	0.971(0.944~0.997)0.032	1.007(0.994~1.021)0.279
Model 2	0.976(0.948~1.004)0.089	1.001(0.987~1.016)0.887
Model 3	0.976(0.948~1.004)0.088	1.001(0.987~1.016)0.891
CRC in male, HR (95% CI), P-value		
Model 1	0.924(0.903~0.944)<0.001	0.978(0.967~0.989)<0.001
Model 2	0.929(0.908~0.951)<0.001	0.976(0.964~0.989)<0.001
Model 3	0.927(0.906~0.949)<0.001	0.976(0.964~0.989)<0.001

Model 1 was adjusted for age, race, educational attainment, and Townsend deprivation index. Model 2, building upon Model 1, adjusted for dietary energy intake; Model 3: In addition to Model 2, adjusted for plasma CRP concentration and NSAIDs medication usage. CRC, Colorectal Cancer; HR, Hazard Ratio; CI, Confidence Interval.

**Table 7:** Leave-One-Out analysis of the oxidative balance score impact on colorectal cancer patients.

	CRC		CRC in female		CRC in male	
	HR(95%CI)	p	HR(95%CI)	p	HR(95%CI)	p
OBS without Carotene	0.971(0.962~0.980)	<0.001	0.995(0.981~1.009)	0.459	0.963(0.952~0.975)	<0.001
OBS without Dietary fiber	0.972(0.963~0.981)	<0.001	0.993(0.980~1.007)	0.349	0.965(0.954~0.977)	<0.001
OBS without Vitamin B6	0.971(0.963~0.980)	<0.001	0.995(0.982~1.009)	0.515	0.962(0.951~0.974)	<0.001
OBS without Total folate	0.971(0.962~0.980)	<0.001	0.995(0.981~1.009)	0.457	0.962(0.951~0.974)	<0.001
OBS without Vitamin B12	0.973(0.965~0.982)	<0.001	0.996(0.983~1.009)	0.561	0.965(0.954~0.976)	<0.001
OBS without Vitamin C	0.971(0.962~0.980)	<0.001	0.994(0.980~1.008)	0.389	0.963(0.952~0.975)	<0.001
OBS without Vitamin E	0.972(0.964~0.981)	<0.001	0.994(0.980~1.007)	0.353	0.964(0.953~0.976)	<0.001
OBS without Calcium	0.974(0.965~0.982)	<0.001	0.996(0.983~1.010)	0.567	0.965(0.954~0.977)	<0.001
OBS without Magnesium	0.973(0.965~0.982)	<0.001	0.995(0.981~1.009)	0.456	0.966(0.955~0.977)	<0.001
OBS without Total fat	0.972(0.964~0.981)	<0.001	0.995(0.982~1.008)	0.416	0.967(0.956~0.978)	<0.001
OBS without Iron	0.975(0.967~0.983)	<0.001	0.995(0.983~1.008)	0.453	0.969(0.959~0.979)	<0.001
OBS without Tea	0.972(0.964~0.980)	<0.001	0.995(0.982~1.009)	0.493	0.964(0.953~0.975)	<0.001
OBS without polyunsaturated fatty acids	0.974(0.965~0.982)	<0.001	0.996(0.983~1.009)	0.567	0.967(0.956~0.978)	<0.001
OBS without saturated fatty acids	0.973(0.965~0.981)	<0.001	0.994(0.982~1.008)	0.410	0.968(0.957~0.978)	<0.001
OBS without vegetable	0.971(0.963~0.979)	<0.001	0.994(0.981~1.008)	0.406	0.964(0.953~0.975)	<0.001
OBS without Retinol	0.975(0.966~0.983)	<0.001	0.995(0.982~1.008)	0.455	0.967(0.956~0.977)	<0.001
OBS without Vitamin D	0.972(0.964~0.980)	<0.001	0.995(0.982~1.008)	0.457	0.963(0.952~0.974)	<0.001
OBS without Meat	0.974(0.966~0.982)	<0.001	0.995(0.982~1.008)	0.472	0.967(0.956~0.978)	<0.001
OBS without Alcohol	0.974(0.965~0.982)	<0.001	0.994(0.981~1.008)	0.398	0.966(0.956~0.978)	<0.001
OBS without Body mass index	0.976(0.968~0.984)	<0.001	0.997(0.984~1.010)	0.659	0.969(0.958~0.980)	<0.001
OBS without Physical activity	0.977(0.969~0.985)	<0.001	0.998(0.986~1.010)	0.685	0.970(0.959~0.980)	<0.001
OBS without Smoking	0.978(0.969~0.986)	<0.001	0.998(0.985~1.012)	0.811	0.970(0.959~0.981)	<0.001

Adjusted for age, race, educational attainment, Townsend deprivation index, dietary energy intake, CRP concentration, dietary energy intake, and NSAIDs medication usage. CRC, Colorectal Cancer; HR, Hazard Ratio; CI, Confidence Interval.

### 3.7 Mediation analysis

**Figure 2:**
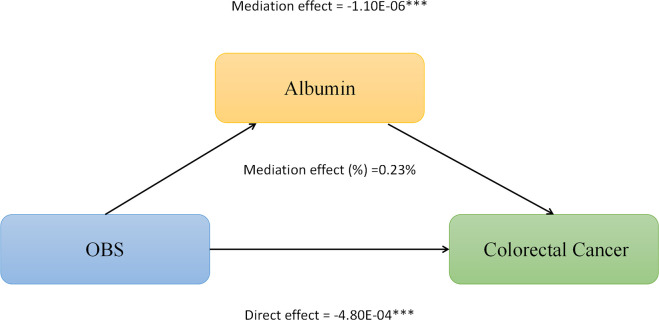
Serum albumin partially mediates the relationship between Oxidative Balance Score (OBS) and colorectal cancer (CRC). ***P<0.001.

**Figure 3:**
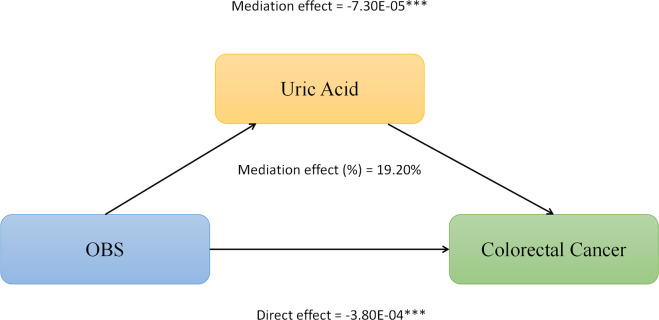
Serum uric acid partially mediates the relationship between Oxidative Balance Score (OBS) and colorectal cancer (CRC). ***P<0.001.

**Figure 4:**
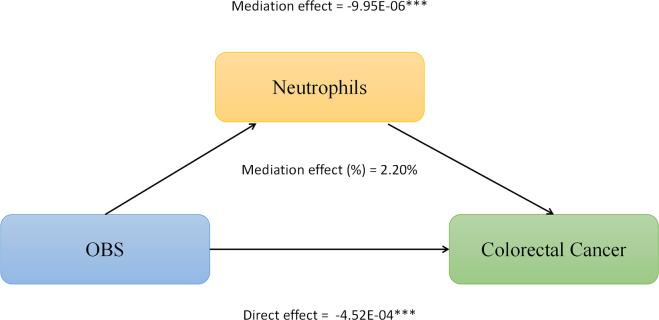
Neutrophils levels partially mediates the relationship between Oxidative Balance Score (OBS) and colorectal cancer (CRC). ***P<0.001.

We conducted mediation analysis to further explore whether serum albumin, uric acid, and neutrophil levels mediate the relationship between OBS and CRC. In this model, OBS was considered as the independent variable, CRC as the dependent variable, and serum albumin, uric acid, and neutrophils as the mediators. As shown in **Figure 2**, serum albumin level mediated the association between OBS and CRC, explaining a total of 0.23% of the variance with a significant mediating effect (IE = -1.10E-06). As shown in **Figure 3**, serum uric acid level mediated the association between OBS and CRC, explaining a total of 19.20% of the variance with a significant mediating effect (IE = -7.30E-05). As shown in **Figure 4**, serum neutrophil level mediated the association between OBS and CRC, explaining a total of 2.20% of the variance with a significant mediating effect (IE = -4.52E-04).

## 4 Discussion

In this large prospective cohort study of the UK Biobank, we found a negative association between higher OBS and the development of CRC, including proximal colon cancer, distal colon cancer, and rectal cancer. In gender-stratified subgroup analysis, a higher dietary OBS and lifestyle OBS were significantly associated with a reduced risk of CRC in male participants, whereas this association was not significant in female participants. Furthermore, there was a positive correlation between the OBS and the risk of CRC metastasis and abdominal pain caused by distal colorectal cancer. On the other hand, there was a negative correlation between the dietary balance score and the risk of CRC metastasis and abdominal pain caused by proximal colorectal cancer. Additionally, we observed a significant correlation between decreased serum albumin levels, increased uric acid levels, increased neutrophil levels, and the occurrence of CRC. Furthermore, we discovered significant mediating effects of serum albumin, uric acid, and neutrophil levels on the relationship between OBS and the risk of CRC, explaining 0.23%, 19.20%, and 2.20% of the correlation, respectively. These findings provide novel insights into the potential biological mechanisms underlying the relationship between OBS indicators and CRC.

Previous studies have found a negative correlation between OBS and the occurrence of sporadic colorectal adenomas and colorectal cancer (10–14). The above studies were mostly conducted in the United States and the Middle East. Due to differences in diet, environment, and other factors, these findings cannot be generalized to the entire population. Our study constitutes the inaugural large-scale cohort study conducted utilizing the UK Biobank dataset, wherein data from 175,808 subjects were analyzed. It is currently one of the largest and most comprehensive biobanks in the world, making the conclusions drawn from this study more accurate and persuasive. According to research by the World Health Organization (WHO), controlling diet and lifestyle can reduce the incidence and mortality rates of cancer, including physical exercise, avoiding smoking, alcohol consumption, and adopting an antioxidant-rich diet (15). Moreover, studies have found that a high intake of whole grains, fruits, vegetables, seafood, nuts, dairy products, and a low intake of red and processed meat can significantly reduce the risk of CRC (2, 3).

The mechanisms by which OBS reduces the risk of CRC can be considered from several aspects: First, OBS aims to assess the balance between oxidative stress and the antioxidant system in the body. Excessive free radicals can exert oxidative stress on the human body, leading to DNA damage, gene mutations, and other issues, thereby increasing the risk of developing cancer (7). When the body’s antioxidant system is effective, it can eliminate these free radicals and prevent damage to DNA, thereby reducing the risk of cancer. Therefore, a higher OBS may reflect better antioxidant capacity, thus reducing the risk of CRC. In addition, oxidative stress can inhibit cell proliferation and promote cell apoptosis through the regulation of pathways such as the nuclear factor kappa B (NF-κB) pathway and mitogen-activated protein kinase (MAPK) pathway (16). In the case of higher OBS, it may promote apoptosis in damaged cells, preventing their development into cancer cells and thus reducing the risk of CRC. The proximal colon has a higher pH and relatively less microbial diversity. The increase in antioxidants and the reduction in oxidative stress may reduce the risk of proximal colon cancer. The distal colon and rectum are closer to the anus, where carcinogens tend to accumulate, thus an elevated OBS may reduce local oxidative stress and lessen the damage to the mucosa of the distal colon and rectum. Simultaneously, improving the health of the gut microbiome could reduce the risk of distal colorectal and rectal cancers (17, 18).

Previous studies have shown a close association between oxidative reactions and inflammatory responses. Maintaining a proper oxidative balance may help control inflammatory responses, thereby preventing inflammation-induced CRC. Our study identified certain circulating biomarkers associated with the risk of CRC, including serum albumin, uric acid, and neutrophils. Albumin is an essential carrier protein for maintaining colloid osmotic pressure. Nutritional deficiencies can reduce the synthesis of albumin in the liver, impairing its levels and functions in the body (19). Hence, low serum albumin levels may indicate compromised nutritional status in the body, often associated with malnutrition or chronic diseases. Low serum albumin levels may lead to decreased immune function, thereby accelerating the growth and spread of cancer cells. Serum uric acid is associated with systemic inflammation and closely linked to defects in the insulin signaling pathway and pancreatic cell dysfunction (20, 21). The increased incidence of CRC due to high uric acid levels may be attributed to the interactions of inflammation, insulin resistance, impaired insulin secretion, and other mechanisms. Elevated levels of neutrophils and activated neutrophils may release inflammatory factors, proteases, and reactive oxygen species, leading to systemic inflammation. Moreover, overactivated neutrophils can also secrete factors that promote angiogenesis and tumor cell migration, directly promoting tumor formation and expansion (22–24).

In this study, dietary OBS and lifestyle OBS were significantly negatively correlated with the occurrence of CRC in males, but not in females. This result may be related to several factors: Firstly, males and females differ in physiological and hormonal levels. Considering the potential protective effect of estrogen on CRC risk (25), we further stratified by age to explore the difference in the impact of OBS on CRC between women aged above and below 50 years. Despite exhaustive stratified analyses, we did not observe a clear association between OBS and CRC risk in women groups aged above and below 50 years, suggesting that the OBS estimate does not take into account the possible estrogen-induced effects on antioxidant capacity. Although our data did not directly prove the interaction between estrogen levels and OBS, existing studies have shown that, estrogen has been shown to resist oxidative stress, inhibit inflammatory responses, prevent DNA damage, promote a more favorable gut microbiota, thereby reducing the risk of CRC (25–27). Therefore, the protective effect of estrogen may make females more resilient to oxidative stress compared to males, even if the OBS score is slightly higher, it may not significantly reduce the risk of CRC in females. Future studies should further investigate how estrogen modulates OBS and its impact on CRC risk. Besides, researches should be extended to larger and more diverse populations to validate these preliminary findings and explore other potential biomarkers. Additionally, there are differences in dietary habits and lifestyles between males and females (28, 29). In the comparison of general characteristics between men and women in this study, males exhibited significantly lower OBS scores than females. Studies also have shown that compared to males, females are more likely to choose a healthy diet in their daily lives, including vegetables, fruits, nuts, etc., which can be considered as powerful sources of antioxidants (30). On the other hand, males may be more exposed to environmental factors that induce oxidative stress, such as excessive alcohol consumption, while females have relatively less exposure (31, 32). Therefore, the healthy diet and lifestyle are more likely to reduce the incidence of CRC in males.

Our study also underscores the importance of considering racial and ethnic diversity in the relationship between dietary and lifestyle-related oxidative balance and CRC risk. In the European ancestry population, the significant inverse association between OBS and CRC risk may suggest that genetic or environmental factors interact differently with oxidative balance compared to other racial groups. Previous research has demonstrated that dietary patterns significantly affect oxidative stress and inflammation, with notable differences across ethnicities that may influence the efficacy of OBS in reducing CRC risk (33). Furthermore, genetic variations among races may alter the biological response to oxidative stress (34). Future studies should aim to confirm these findings in a larger and more diverse population sample and further explore the potential mechanisms by which racial differences affect the impact of OBS on CRC risk.

Our study found that an elevated OBS reduces the risk of secondary metastasis and abdominal pain caused by proximal colon cancer, yet it elevates the risk of secondary metastasis and abdominal pain induced by distal colon cancer. This may be due to the fact that differences in genetic and molecular characteristics of proximal colon cancer result in antioxidants having a more positive effect, such as reducing DNA damage caused by oxidative stress, thereby lowering the risk of metastasis and symptoms induced by the tumor (35). Some antioxidants may promote the proliferation and metastasis of existing tumor cells at high doses, especially when the tumor cells have adapted to a high oxidative stress environment (36). The balance between antioxidants and pro-oxidants may affect different sites of colon cancer differently, possibly related to the biological characteristics of the tumor, the mechanism of action of antioxidants in different tissues, and individual genetic differences (37). Therefore, future studies will need more in-depth, large-scale prospective clinical trials and biological researches to further explore the complex interactions between biomarkers such as the OBS score and tumor cells and tissue.

Our study has several limitations. First, the design of a cross-sectional study limits causal inferences between OBS and CRC, and further prospective cohort studies are needed to investigate the causal relationship between OBS and CRC. Second, our study collected various components of OBS using self-reported FFQ questionnaires, which may result in partial recall bias. Furthermore, our study lacks exploration of epigenetic and environmental factors that may interact with OBS indicators. Future research should include more studies on genetics and metabolomics to further investigate the antioxidant mechanisms underlying the relationship between OBS and CRC occurrence.

Despite these limitations, our study still has several notable strengths. Firstly, this study provides robust evidence for the utility of OBS indicators in the risk assessment of CRC and its subsites in a large population cohort in the UK. These findings may guide clinical decisions related to CRC and further research is needed to validate the predictive performance of dietary and lifestyle OBS in different cohorts and environments. Moreover, the participant data in this study were obtained from the large-scale UK Biobank database, a long-term, population-based prospective study, which has a massive sample size that effectively minimizes sampling bias. Furthermore, our study highlights the potential role of oxidative stress in CRC and its subsites. OBS, as a composite measure based on oxidative balance, can more accurately reflect the oxidative and antioxidant status in the body compared to individual biomarkers. Our findings provide several guiding directions for future research. Firstly, exploring the impact of genetic and metabolic factors, demographics, lifestyle, and other factors on the association between OBS and CRC and its subsites. This will help to further develop and evaluate strategies for CRC screening and prevention based on OBS. Furthermore, employing more comprehensive and specific methods such as mediation analysis and machine learning to further characterize the dose-response relationship between OBS and CRC would be beneficial for clinicians to explore optimizing antioxidant status through diet, supplements, and lifestyle for improved prognosis of CRC patients.

## 5 Conclusion

In summary, this large prospective cohort study demonstrates a significant inverse association between OBS and the risk of CRC and its subsites, with a stronger association observed in the male CRC population. Serum albumin, uric acid, and neutrophils partially mediate the association between OBS and CRC. Our study results emphasize that antioxidant-rich diet and lifestyle may serve as targets for the prevention and treatment of CRC. Further research is needed to elucidate the complex interactions between diet, lifestyle, relevant biomarkers, and the occurrence of CRC.

## Data availability statement

The dataset from the UK Biobank can be accessed by researchers through the application process at http://www.ukbiobank.ac.uk/regis. The Application ID for this dataset is 84347.

## Ethics statement

The studies involving humans were approved by The North West Multi-center Research Ethics Committee, Manchester, U.K. (REC reference for UK Biobank 11/NW/0382). The studies were conducted in accordance with the local legislation and institutional requirements. Written informed consent for participation in this study was provided by the participants’ legal guardians/next of kin.

## Author contributions

YC: Data curation, Formal analysis, Methodology, Software, Visualization, Writing – original draft, Writing – review & editing. FL: Data curation, Project administration, Software, Visualization, Writing – original draft. ZhiW: Investigation, Validation, Writing – original draft. QZ: Investigation, Validation, Writing – original draft. ZDW: Investigation, Validation, Writing – original draft. XH: Investigation, Validation, Writing – original draft. ZhaW: Investigation, Validation, Writing – original draft. CY: Investigation, Validation, Writing – original draft. YL: Investigation, Validation, Writing – original draft. SC: Investigation, Validation, Writing – original draft. HL: Investigation, Validation, Writing – original draft. SH: Investigation, Validation, Writing – original draft. YQL: Resources, Supervision, Writing – review & editing. TT: Resources, Supervision, Writing – review & editing.
