# Supplementary material for: Oxidative balance score: a potential tool for reducing the risk of colorectal cancer and its subsites incidences
Source: Front Endocrinol (Lausanne). 2024 Apr 30;15:1397512. doi: 10.3389/fendo.2024.1397512 (PMC11091465; doi:10.3389/fendo.2024.1397512)
Supplement: Supplementary file 1 [file Table_1.docx]

Supplementary Material

Supplemental Table 1. Components of the Oxidative Balance Score Stratified by Sex

| OBS components | Unit | Property | Category | Female | | | Male | | |
| --- | --- | --- | --- | --- | --- | --- | --- | --- | --- |
|  |  |  |  | 0 | 1 | 2 | 0 | 1 | 2 |
| Total fat | g/day | P | Nutrient Intake | >82.15 | 56.19~82.15 | <56.19 | >94.27 | 64.54~94.27 | <64.54 |
| Iron | mg/day | P | Nutrient Intake | >14.59 | 10.77~14.59 | <10.77 | >16.26 | 12~16.26 | <12 |
| polyunsaturated fatty acids | g/day | P | Nutrient Intake | >15.5 | 8.89~15.5 | <8.89 | >17.59 | 10.08~17.59 | <10.08 |
| saturated fatty acids | g/day | P | Nutrient Intake | >31.32 | 20.66~31.32 | <20.66 | >36.36 | 23.91~36.36 | <23.91 |
| Meat | times/week | P | Lifestyle | >3 | >0 & <3 | =0 | >3 | >0 &<3 | =0 |
| Carotene | ug/day | A | Nutrient Intake | <1370.22 | 1370.22~4147.08 | >4147.08 | <1014.02 | 1014.02~3374.59 | >3374.59 |
| Dietary fiber | g/day | A | Nutrient Intake | <12.78 | 12.78~18.49 | >18.49 | <12.99 | 12.99~19.13 | >19.13 |
| Vitamin B6 | mg/day | A | Nutrient Intake | <1.72 | 1.72~2.37 | >2.37 | <1.87 | 1.87~2.58 | >2.58 |
| Total folate | ug/day | A | Nutrient Intake | <233.04 | 233.04~326.78 | >326.78 | <253.96 | 253.96~354.33 | >354.33 |
| Vitamin B12 | mg/day | A | Nutrient Intake | <3.49 | 3.49~6.48 | >6.48 | <3.77 | 3.77~6.76 | >6.76 |
| Vitamin C | mg/day | A | Nutrient Intake | <91.45 | 91.45~178.59 | >178.59 | <82.29 | 82.29~168.05 | >168.05 |
| Vitamin E | mg/day | A | Nutrient Intake | <6.67 | 6.67~10.46 | >10.46 | <6.42 | 6.42~10.37 | >10.37 |
| Calcium | mg/day | A | Nutrient Intake | <749.84 | 749.84~1049.64 | >1049.64 | <803.12 | 803.12~1122.7 | >1122.7 |
| Magnesium | mg/day | A | Nutrient Intake | <280.59 | 280.59~365.89 | >365.89 | <308.35 | 308.35~404.4 | >404.4 |
| Retinol | ug/day | A | Nutrient Intake | <198.99 | 198.99~359.63 | >359.63 | <222.51 | 222.51~407.21 | >407.21 |
| Vitamin D | ug/day | A | Nutrient Intake | <1.03 | 1.03~2.45 | >2.45 | <1.2 | 1.2~2.76 | >2.76 |
| Tea | cups/day | A | Lifestyle | <2 | 2~4 | >4 | <2 | 2~4 | >4 |
| vegetable | tablespoons/day | A | Lifestyle | <4 | 4~6 | >6 | <3 | 3~5 | >5 |
| Alcohol | g/day | P | Lifestyle | >19.52 | >0 & <19.52 | =0 | >31.32 | >0 &<31.32 | =0 |
| BMI | kg/m2 | P | Lifestyle | >28.30 | 24.34~28.30 | <24.34 | >29.00 | 25.79~29.00 | <25.79 |
| Physical activity | MET minutes/week | A | Lifestyle | <1062 | 1062~2685.17 | >2685.17 | <1108.5 | 1108.5~2897.17 | >2897.17 |
| Smoking | package years | P | Lifestyle | >17 | >0 & <17 | =0 | >21.5 | >0 &<21.5 | =0 |

Notes: OBS, Oxidative Balance Score; A, antioxidant; P, pro-oxidant; MET, metabolic equivalent of task; BMI, body mass index

Supplemental Table 2. Baseline Characteristics of Participants Stratified by Sex

| Characteristic | Overall N = 175,808 | Female N = 94,044 | Male N = 81,764 | p-value |
| --- | --- | --- | --- | --- |
| OBS, Mean (SD) | 22.5 (5.0) | 22.6 (5.1) | 22.3 (5.0) | <0.001 |
| TDI, Mean (SD) | -1.6 (2.9) | -1.5 (2.8) | -1.6 (2.9) | <0.001 |
| Missing | 221 | 108 | 113 |  |
| Education, Mean (SD), score | 11.7 (13.8) | 11.6 (13.6) | 11.8 (13.9) | 0.006 |
| Missing | 4,482 | 2,354 | 2,128 |  |
| Ethnicity, n (%) |  |  |  | <0.001 |
| European | 167,952 (96%) | 89,748 (96%) | 78,204 (96%) |  |
| Mixed-race | 1,054 (0.6%) | 667 (0.7%) | 387 (0.5%) |  |
| Asian | 2,488 (1.4%) | 1,155 (1.2%) | 1,333 (1.6%) |  |
| African | 2,082 (1.2%) | 1,239 (1.3%) | 843 (1.0%) |  |
| Chinese | 495 (0.3%) | 315 (0.3%) | 180 (0.2%) |  |
| Others | 1,239 (0.7%) | 734 (0.8%) | 505 (0.6%) |  |
| Missing | 498 | 186 | 312 |  |
| BMI, Mean (SD), Kg/m2 | 26.9 (4.6) | 26.4 (4.9) | 27.4 (4.1) | <0.001 |
| Age, Mean (SD), years | 55.9 (8.0) | 55.3 (7.9) | 56.5 (8.1) | <0.001 |
| CRP level, Mean (SD), mg/L | 2.3 (4.0) | 2.3 (3.9) | 2.2 (4.0) | <0.001 |
| Missing | 10,192 | 5,674 | 4,518 |  |
| Colorectal cancer, n (%) | 2,626 (1.5%) | 1,061 (1.1%) | 1,565 (1.9%) | <0.001 |
| Proximal colon cancer, n (%) | 786 (0.4%) | 354 (0.4%) | 432 (0.5%) | <0.001 |
| Distal colon cancer, n (%) | 786 (0.4%) | 318 (0.3%) | 468 (0.6%) | <0.001 |
| Rectal cancer, n (%) | 757 (0.4%) | 262 (0.3%) | 495 (0.6%) | <0.001 |
| Daily energy intake, Mean (SD) | 8,862.8 (3,036.6) | 8,239.4 (2,751.7) | 9,579.8 (3,186.9) | <0.001 |
| Death of CRC, n (%) | 509 (0.3%) | 189 (0.2%) | 320 (0.4%) | <0.001 |
| NSAIDs medication usage, n (%) | 59,478 (34%) | 32,182 (34%) | 27,296 (33%) | <0.001 |
| CRC with obstruction, n (%) | 275 (0.2%) | 98 (0.1%) | 177 (0.2%) | <0.001 |
| CRC with abdominal pain, n (%) | 294 (0.2%) | 131 (0.1%) | 163 (0.2%) | 0.008 |
| Secondary Metastasis of CRC, n (%) | 662 (0.4%) | 243 (0.3%) | 419 (0.5%) | <0.001 |
| Albumin, Mean (SD), g/L | 45.4 (2.6) | 45.1 (2.6) | 45.7 (2.6) | <0.001 |
| Missing | 24,201 | 13,847 | 10,354 |  |
| Uric acid, Mean (SD), umol/L | 306.4 (79.1) | 266.0 (63.2) | 352.5 (69.8) | <0.001 |
| Missing | 10,035 | 5,626 | 4,409 |  |
| Neutrophils, Mean (SD), 10^9 cells/Litre | 4.1 (1.4) | 4.1 (1.3) | 4.1 (1.4) | <0.001 |
| Missing | 6,810 | 4,003 | 2,807 |  |

Notes: OBS, Oxidative Balance Score; TDI, Thomson Deprivation Index; CRC, Colorectal Cancer; SD, standard deviation; BMI, body mass index; CRP, C-reactive protein; Education refered to the age of Highest Level of Education

Supplemental Table 3. The Relationship Between OBS and CRC Incidence Risk Among Women Stratified by Age Group

|  |  | HR 95%CI | P-value |
| --- | --- | --- | --- |
| Female, age>50 | Model 1 | 0.998(0.985~1.011) | 0.785 |
|  | Model 2 | 0.992(0.979~1.006) | 0.267 |
|  | Model 3 | 0.992(0.979~1.006) | 0.272 |
| Female, age≤50 | Model 1 | 1.012(0.978~1.047) | 0.479 |
|  | Model 2 | 1.016(0.980~1.054) | 0.394 |
|  | Model 3 | 1.015(0.979~1.053) | 0.424 |

Notes: Model 1 was adjusted for age, race, educational attainment, and Townsend deprivation index. Model 2, building upon Model 1, adjusted for dietary energy intake; Model 3: In addition to Model 2, adjusted for plasma CRP concentration and NSAIDs medication usage. HR, Hazard Ratio; CI, Confidence Interval.

Supplemental Table 4. The Relationship Between OBS and CRC Incidence Risk Stratified by Racial Groups

| Race/Ethnicity |  | HR 95%CI | P-value |
| --- | --- | --- | --- |
| European | Model 1 | 0.978(0.971~0.986) | <0.001 |
|  | Model 2 | 0.973(0.965~0.981) | <0.001 |
|  | Model 3 | 0.973(0.965~0.981) | <0.001 |
| Mixed-race | Model 1 | 1.058(0.965~1.159) | 0.227 |
|  | Model 2 | 1.055(0.955~1.165) | 0.291 |
|  | Model 3 | 1.050(0.954~1.165) | 0.290 |
| Asian | Model 1 | 0.930(0.841~1.029) | 0.159 |
|  | Model 2 | 0.911(0.813~1.020) | 0.106 |
|  | Model 3 | 0.907(0.809~1.018) | 0.097 |
| African | Model 1 | 0.980(0.904~1.063) | 0.622 |
|  | Model 2 | 1.009(0.920~1.106) | 0.856 |
|  | Model 3 | 1.013(0.923~1.111) | 0.791 |
| Chinese | Model 1 | 1.249(0.966~1.614) | 0.090 |
|  | Model 2 | 1.113(0.822~1.509) | 0.489 |
|  | Model 3 | 1.113(0.810~1.530) | 0.509 |
| Others | Model 1 | 0.992(0.892~1.103) | 0.882 |
|  | Model 2 | 1.013(0.902~1.137) | 0.831 |
|  | Model 3 | 1.009(0.899~1.132) | 0.884 |

Notes: Model 1 was adjusted for age, race, educational attainment, and Townsend deprivation index. Model 2, building upon Model 1, adjusted for dietary energy intake; Model 3: In addition to Model 2, adjusted for plasma CRP concentration and NSAIDs medication usage. HR, Hazard Ratio; CI, Confidence Interval.
